# Insights from Alzheimer’s Disease Multimodal Screening and Prevention Strategies in Egyptian Healthcare Institutions: A Systematic Literature Review

**DOI:** 10.1002/alz.087515

**Published:** 2025-01-09

**Authors:** Youssef Haitham, Youssef A. Ismail, Mohammad Walid, Hazim Mohamed, Youssef M. Abd El‐Satar

**Affiliations:** ^1^ STEM Neurology & Neuropsychological0 Research Group Egypt (SNRGE), Port Said, Port Said Egypt; ^2^ Faculty of Medicine Suez Canal Univeristy, Egypt, Ismailia, Ismailia Egypt; ^3^ Faculty of Medicine Port Said Univeristy, Egypt, Port Said, Port Said Egypt; ^4^ Faculty of Medicine, Arish University, Arish, North Sinai Egypt; ^5^ Faculty of Medicine Helwan University, Cairo, Helwan Egypt; ^6^ Faculty of Medicine Cairo Univeristy, Egypt, Cairo, Cairo Egypt

## Abstract

**Background:**

Alzheimer’s Disease (AD) is a progressive, incurable neurological condition that is the most common cause of dementia. Five percent of people aged 65 to 74, 13.1% of people aged 75 to 84, and 33.3% of people aged 85 or older have AD, according to 2023 statistics. Due to the lack of scientific data comparing the success and cost‐effectiveness of screening programs and trial recruiting strategies, the main goal of this paper is to determine the existence and quality of AD screening (if existed) in Egypt, including how often it’s done, how common it is and how much information is known about the importance of AD screening among both healthcare professionals and the general public.

**Methods:**

We reviewed database searches through November 2023 in MEDLINE, PsycINFO, and the Cochrane Central Register of Controlled Trials; and additional searches for ongoing trials through ClinicalTrials.gov, World Health Organization International Clinical Trials Registry Platform, and Current Controlled Trials (ISRCTN Register). We also investigated healthcare units in *Port Said*, *Ismailia* and some units in *Cairo* about typical AD screening programs that have been established or scheduled to be initiated in the future.

**Results:**

Healthcare units investigations show that the AD screening program has not been established yet and isn’t scheduled for any future dates. There was no previous history of screening programs being held in Egypt in any digital database. AD screening programs are a crucial part of regular public health awareness, especially for elderly individuals that are particularly vulnerable to its effects and are affected by it at a disproportionately high level compared to other people.

**Conclusion:**

We could not find any solid evidence on the previous existence of any AD screening programs in Egypt. Further investigations are required to drive conclusive decisions about the quality of screening programs in Egypt.

